# Signal-in-space range error and positioning accuracy of BDS-3

**DOI:** 10.1038/s41598-022-12012-y

**Published:** 2022-05-17

**Authors:** Weiping Liu, Bo Jiao, Jinming Hao, Zhiwei Lv, Jiantao Xie, Jing Liu

**Affiliations:** Information Engineering University, Zhengzhou, 450001 China

**Keywords:** Geodynamics, Geomorphology

## Abstract

Being the first mixed-constellation global navigation system, the global BeiDou navigation system (BDS-3) designs new signals, the service performance of which has attracted extensive attention. In the present study, the Signal-in-space range error (SISRE) computation method for different types of navigation satellites was presented. The differential code bias (DCB) correction method for BDS-3 new signals was deduced. Based on these, analysis and evaluation were done by adopting the actual measured data after the official launching of BDS-3. The results showed that BDS-3 performed better than the regional navigation satellite system (BDS-2) in terms of SISRE. Specifically, the SISRE of the BDS-3 medium earth orbit (MEO) satellites reached 0.52 m, slightly inferior compared to 0.4 m from Galileo, marginally better than 0.59 m from GPS, and significantly better than 2.33 m from GLONASS. The BDS-3 inclined geostationary orbit (IGSO) satellites achieved the SISRE of 0.90 m, on par with that (0.92 m) of the QZSS IGSO satellites. However, the average SISRE of BDS-3 geostationary earth orbit (GEO) satellites was 1.15 m, which was marginally inferior to that of the QZSS GEO satellite (0.91 m). In terms of positioning accuracy, the new signals B1C and B2a are considered together with the transition signals B1I and B3I. The overall three-dimensional single-frequency standard point positioning (SPP) accuracy of BDS-3 B1C, B2a, B1I, and B3I gained an accuracy level better than 5 m. Moreover, the B1I signal exhibited the best positioning accuracy in the Asian-Pacific region, while the B1C signal set forth the best positioning accuracy in the other regions. Owing to the advantage in signal frequency, the dual-frequency SPP accuracy of B1C + B2a surpassed that of the transitional signal of B1I + B3I. Since there are more visible satellites in Asia–Pacific, the positioning accuracy of BDS-3 was moderately superior to that of GPS. The precise point positioning (PPP) accuracy of BDS-3 B1C + B2a or B1I + B3I converged to the order of centimeters, marginally inferior to that of the GPS L1 + L2. However, these three combinations had a similar convergence time of approximately 30 min.

## Introduction

**Figure 1:**
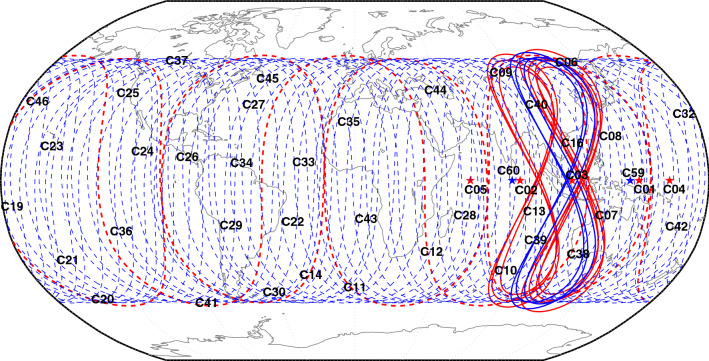
Tracks of sub-satellite points for in-orbit operational BeiDou satellites. The red and blue lines denote BDS-2 and BDS-3 satellites, respectively.

According to a steady “three-step” strategy^1^, BeiDou Navigation Satellite System (BDS) is independently established and operated by China. On July 31st, 2020, based on the demonstration navigation satellite system (BDS-1) and the regional navigation satellite system (BDS-2), the global BeiDou navigation system (BDS-3) was officially announced as operational. BDS-3 comprises three geostationary earth orbit (GEO) satellites, three inclined geostationary orbit (IGSO) satellites, and 24 medium earth orbit (MEO) satellites^2^. All these satellites have been providing services normally except for the last launched GEO satellite. Considering that BDS-2 still has five GEO satellites, seven IGSO satellites, and three MEO satellites that are functioning normally in orbit, the current BeiDou System has a total of 44 operational satellites in orbit that can provide services. The corresponding satellite types are listed in Table 1. The tracks of sub-satellite points are illustrated in Fig. 1, in which the red line represents the BDS-2 satellites while the blue indicates the BDS-3 satellites. BDS-3 adopts the new signals B1C (1575.42 MHz) and B2a (1176.45 MHz) for open service, which are broadcast on the BDS-3 MEO and IGSO satellites^3–5^. Concurrently, for smooth transitioning with BDS-2, all types of BDS-3 satellites allow for compatible broadcasting of B1I (1561.098 MHz) and B3I (1207.14 MHz)^6,7^, as mentioned in Table 2. The frequency difference between these two new signals B1C and B2a is 398.97 MHz, which is greater than 353.958 MHz of those two signals B1I and B3I. When the new signals form ionosphere-free combination, it will be more conducive to the elimination of ionospheric delay error. A series of improvements have been inculcated in the ground segment of BDS-3^8^, and the inter-satellite link is added at its space segment^9^. The performance of the ground segment and the space segment demands in-depth analysis and evaluation. Besides, the positioning performance of the new signals and the transitional signals is a matter of concern for the user segment.

**Table 1:** Operational BeiDou System satellites in orbit. The last launched BDS-3 GEO satellite C61 is not included, because it is still in the in-orbit testing phase when the paper is completed.

System	Satellite type	PRN
BDS-2	GEO	C01-C05
	IGSO	C06-C10, C13, C16
	MEO	C11, C12, C14
BDS-3	GEO	C59, C60
	IGSO	C38-C40
	MEO	C19-C30, C32-C37, C41-C46

**Table 2:** Open service signals of BeiDou System. B1C and B2a are the new signals of BDS-3, while B1I and B3I are the compatible signals of both BDS-2 and BDS-3.

Signal	Center frequency (MHz)	Broadcasting satellites
B1C	1575.42	BDS-3: 3IGSO + 24MEO
B2a	1176.45	BDS-3: 3IGSO + 24MEO
B1I	1561.098	BDS-2: 5GEO + 7IGSO + 3MEO BDS-3: 3GEO + 3IGSO + 24MEO
B3I	1268.52	BDS-2: 5GEO + 7IGSO + 3MEO BDS-3: 3GEO + 3IGSO + 24MEO

On the one hand, the accuracy of user positioning is affected by the spatial geometric distribution of navigation satellites, which has been basically determined in the design phase of satellite navigation system. On the other hand, it is affected by the user equivalent range error (UERE), which can be divided into the user equipment error (UEE) and the signal-in-space range error (SISRE). The UEE mainly reflects the errors related to user reception, including noise, multipath, uncorrected ionospheric delay and tropospheric delay, etc. The SISRE reflects the error of the broadcast ephemeris orbit and clock offset, which primarily showcases the performance of the space segment and the ground segment. Montenbruck et al. (2015) pointed out that the SISRE could be used as a key system indicator to analyze the overall performance of the ground segment and the space segment^10^. The commonly used method is to select the precise orbit and clock offset as the standard to evaluate the accuracy level of the broadcast ephemeris orbit and clock offset, and then obtain the SISRE. With the development of BeiDou, many scholars conducted analysis and research on the SISRE of the system. Considering the data from March 2013 to September 2016, Wu et al. (2017) took the precise orbit and clock offset as the standard to obtain a detailed analysis of the evolution of the BDS-2 SISRE^11^. In 2018, Montenbruck et al*.* (2018) employed the multi-system broadcast ephemeris data to compare the SISRE of the satellite navigation systems such as GPS, GLONASS, Galileo and BDS-2^12^. And then, Ouyang et al. (2019) analyzed BDS-2 broadcast navigation message from 2013 to 2018^13^. In 2020, Yang et al*.* (2020) and Chen et al. (2020) respectively examined the SISRE of 18 MEO satellites of the basic BDS-3 constellation, validating the satisfactory performance of BDS-3 and proposing technological assumptions for future developments of the system^14,15^. In the same year, Montenbruck et al. (2020) studied the difference in SISRE for GPS, GLONASS, Galileo, and BDS-3 (only considering BDS-3 MEO satellites)^16^, and Jiao et al. (2020) compared BDS-3 and BDS-2 broadcast ephemeris^17^. In 2021, Xue et al. (2021) mainly analyzed the influence of clock errors on BDS-3 SISRE base on the data from January 1 to December 31 in the year of 2019^18^, and Chen et al. (2021) made a further comparison between BDS-3 SISRE and BDS-2 SISRE^19^. However, the existing studies mainly discuss the SISRE of BDS-3 MEO satellites in the basic BDS-3 constellation, but there is little discussion on the newly launched MEO satellites, especially IGSO and GEO satellites. In addition, there is a lack of comparison between the completed BDS-3 and other satellite navigation systems.

Besides, the level of positioning accuracy, a key performance indicator for the BeiDou System, has long been valued. Since the time BDS-2 began to operate, there have been plenty of articles about the systemic analysis on its positioning performance^20–23^. Later, with the construction and development of BDS-3, multiple studies have evaluated the level of positioning accuracy. With the five experimental BDS-3 satellites, Yang et al. (2018) and Zhang et al. (2017) analyzed the observation quality of the BDS-3 new signals in terms of carrier-to-noise ratio, multipath, and observed quantity combination^24,25^. Zhang et al*.* (2019) conducted a preliminary assessment about the signal quality of BDS-3 and the positioning performance of Real-Time Kinematic (RTK) and Precise Point Positioning (PPP)^26^. Shi et al. (2020) and Lv et al. (2019) considered eighteen MEO satellites and one GEO satellite from the basic constellation of BDS-3 to analyze the positioning performance^27,28^. However, the above analyses were mostly conducted when the construction of BDS-3 was incomplete. Moreover, the assessment for the full constellation positioning performance of the BDS-3 new signals was limited.

We here present the SISRE computation method for different types of navigation satellites and deduce the differential code bias (DCB) correction method for BDS-3 new signals during the positioning data processing. Then the SISRE of BDS-3 is analyzed including GEO, IGSO, and MEO satellites. Also, the SISREs of BDS-3, BDS-2, GPS, GLONASS, Galileo, and QZSS are compared by processing measured data. Meanwhile, Standard Point Positioning (SPP) and PPP, the two positioning modes that cannot eliminate the common errors by observation difference and can best reflect the data quality of the pseudo-code and the carrier phase, are adopted to perform the in-depth analysis on the positioning performance of the new signals and the transitional signals for BDS-3.

## Methods

Within this section, SISRE computation method for different types of satellites is introduced, which is convenient to compute and compare the SISREs of different navigation systems. To evaluate performance of BDS-3 SPP and PPP, the DCB correction method for B1C and B2a signals is deduced.

### SISRE

The SISRE, which involves two parts, i.e., the satellite orbit error and the satellite clock offset error, is evaluated as follows. The multi-system precise orbit and clock offset, which are calculated by the International GNSS Service (IGS) analysis centers, are taken as the standard and the broadcast ephemeris is used to derive the satellite orbit and the clock offset of the corresponding epoch, analyzing their difference to gain the SISRE. During the analysis, some issues should be processed, such as the antenna phase offset correction, the time group delay, et al^12,14,16^. In order to enhance comparison effects, the following work has been done: first of all, distinguish three different satellite types such as GEO, IGSO and MEO to reflect the SISRE differences in the two cases for different satellite types of the same system and the same satellite type of different systems; Secondly, some major navigation systems such as BDS, GPS, Galileo, GLONASS, and QZSS are simultaneously considered for mutual comparison; Finally, a long period of data is analyzed to enhance the credibility of the analysis conclusion.

The computation equation of SISRE for different types of satellites is as follows:

1 $$SISRE = \sqrt {(w_{R}^{{}} \cdot R - c\delta t)^{2} + w_{A,C}^{2} \cdot (A^{2} + C^{2} )}$$

where $w_{R}^{{}}$ and $w_{A,C}^{{}}$ are corresponding weight factors that are related to the satellite altitude^10^, as mentioned in Table 3; $R$, $A$, and $C$ denote the radial, along-track, and cross-track, respectively; $\delta t$ indicates the clock offset error of the satellite; and $c$ represents the speed of light.

**Table 3:** Values of weight factors for different systems. These values are mainly related to the satellite altitude.

System *w*_*R*_	*w*_*R*_	$w_{A,C}^{2}$
BDS (MEO)	0.98	1/54
BDS (IGSO, GEO)	0.99	1/126
GPS	0.98	1/49
GLONASS	0.98	1/45
Galileo	0.98	1/61
QZSS (IGSO, GEO)	0.99	1/126

### SPP and PPP processing for BDS-3

SPP and PPP have been elaborated extensively in the literature. The chief issue of the DCB correction is only explained here when processing the new signals of B1C and B2a for BDS-3. The reference signal of the clock offset in BeiDou broadcast ephemeris is B3I, whereas the precise clock offset products are typically calculated by the B1I and B3I ionosphere-free combination. Hence, whether the broadcast ephemeris or the precise product is in use while processing B1C or B2a, the DCB correction should be noted.

Here, the B1C and B2a ionosphere-free combination for PPP is taken as an example. In this case, the B1C and B2a dual-frequency pseudo-code ionosphere-free combination^4^ should be utilized as:

2 $$PC_{B1C\_B2a} = R + c\left( {\delta t_{r} - \delta t_{s} } \right) + \delta_{trop} + c\left( {{{\left( {f_{B1C}^{2} \tau_{B1C} - f_{B2a}^{2} \tau_{B2a} } \right)} \mathord{\left/ {\vphantom {{\left( {f_{B1C}^{2} \tau_{B1C} - f_{B2a}^{2} \tau_{B2a} } \right)} {\left( {f_{B1C}^{2} - f_{B2a}^{2} } \right)}}} \right. \kern-\nulldelimiterspace} {\left( {f_{B1C}^{2} - f_{B2a}^{2} } \right)}}} \right)$$

where $PC_{B1C\_B2a}$ indicates the B1C and B2a dual-frequency pseudo-code ionosphere-free combination observation; $R$ indicates the geometrical distance between the receiver and the satellite; $c$ denotes the speed of light; $\delta t_{r}$ and $\delta t_{s}$ represent the receiver clock offset and the satellite clock offset, respectively; $\delta_{trop}$ is the tropospheric correction; $f_{B1C}^{{}}$ and $f_{B2a}^{{}}$ denote the frequency of B1C and B2a, respectively; $\tau_{B1C}$ and $\tau_{B2a}$ denote the internal signal delay of the B1C and the B2a pseudo-code, respectively.

According to (2), the satellite clock offset and the internal signal delay cannot be separated. The satellite clock offset $\delta t_{st(B1C\_B2a)}$ that includes the internal signal delay is defined as:

3 $$\delta t_{st(B1C\_B2a)} = \delta t_{s} - {{\left( {f_{B1C}^{2} \tau_{B1C} - f_{B2a}^{2} \tau_{B2a} } \right)} \mathord{\left/ {\vphantom {{\left( {f_{B1C}^{2} \tau_{B1C} - f_{B2a}^{2} \tau_{B2a} } \right)} {\left( {f_{B1C}^{2} - f_{B2a}^{2} } \right)}}} \right. \kern-\nulldelimiterspace} {\left( {f_{B1C}^{2} - f_{B2a}^{2} } \right)}}$$

Similarly, the precise clock offset of BeiDou is often derived through the B1I and B3I ionosphere-free combination. We have

4 $$\delta t_{st(B1I\_B3I)} = \delta t_{s} - {{\left( {f_{B1I}^{2} \tau_{B1I} - f_{B3I}^{2} \tau_{B3I} } \right)} \mathord{\left/ {\vphantom {{\left( {f_{B1I}^{2} \tau_{B1I} - f_{B3I}^{2} \tau_{B3I} } \right)} {\left( {f_{B1I}^{2} - f_{B3I}^{2} } \right)}}} \right. \kern-\nulldelimiterspace} {\left( {f_{B1I}^{2} - f_{B3I}^{2} } \right)}}$$

Here, $\delta t_{st(B1I\_B3I)}$ denotes the satellite clock offset that contains the internal signal delay of the B1I and B3I ionosphere-free combination; $f_{B1I}^{{}}$ and $f_{B3I}^{{}}$ denote the frequency of B1I and B3I, respectively; $\tau_{B1I}$ and $\tau_{B3I}$ represent the internal signal delay of the B1I and the B3I pseudo-code, respectively; $\delta t_{s}$ is the satellite clock offset as in (2) and (3).

According to (3) and (4), the relationship between $\delta t_{st(B1C\_B2a)}$ and $\delta t_{st(B1I\_B3I)}$ can be derived. We have

5 $$\begin{aligned} \delta t_{st(B1C\_B2a)} & = \delta t_{st(B1I\_B3I)} + \left\{ {(f_{B1I}^{2} \cdot f_{B1C}^{2} \cdot (\tau_{B1I} - \tau_{B1C} ) + } \right. \, f_{B1C}^{2} \cdot f_{B3I}^{2} \cdot (\tau_{B1C} - \tau_{B3I} ) \\ & \quad + \, f_{B1I}^{2} \cdot f_{B2a}^{2} \cdot (\tau_{B2a} - \tau_{B1I} ) - f_{B3I}^{2} \cdot f_{B2a}^{2} \cdot (\tau_{B2a} - \tau_{B3I} ){ )}/\left. {((f_{B1I}^{2} - f_{B3I}^{2} ) \cdot (f_{B1C}^{2} - f_{B2a}^{2} ))} \right\} \\ \end{aligned}$$

Here, $(\tau_{B1I} - \tau_{B1C} )$, $(\tau_{B1C} - \tau_{B3I} )$, $(\tau_{B2a} - \tau_{B1I} )$, and $(\tau_{B2a} - \tau_{B3I} )$ can be derived from the DCB correction data released by the related organizations which is downloaded from ftp://igs.ign.fr/pub/igs/products/mgex/dcb in the following analysis. Therefore, while using the precise clock offset of BeiDou for the PPP processing of the B1C and B2a ionosphere-free combination, it is mandatory to perform the DCB correction, as represented by “{}” in (5). In SPP processing, the similar problem need to be addressed.

## Results

In this section, the actual measured data was analyzed after the official launching of the BDS-3 service. At first, the SISRE of BDS-3 was computed and compared to that of BDS-2, GPS, GLONASS, Galileo and QZSS. And then, the positioning performance of BDS-3 was analyzed in both cases of SPP and PPP. In the analysis, special attention was paid to the new signals of BDS-3.

### SISRE computation

According to the SISRE computation method specified above, the broadcast ephemeris was first used to compute the satellite position and the clock offset of 96 epochs with an interval of 900 s for each day. Later, considering the precise ephemeris and the precise clock offset at the corresponding epoch as the standard, the errors of the orbit and the clock offset were evaluated. Based on this, the SISRE was calculated. The multi-system BRDM long-filename file in the receiver independent exchange format (RINEX) provided by Multi-GNSS Experiment (MGEX) was adopted as the broadcast ephemeris. The GBM precise product was adopted as the precise orbit and the precise clock offset respectively with a sampling interval of 300 s and 30 s.

**Figure 2:**
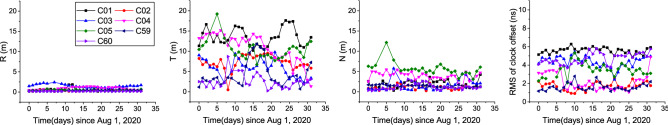
Orbit and clock offset accuracy variations of the GEO satellites. From left to right: Radial, tangential, normal, satellite clock offset.

**Figure 3:**
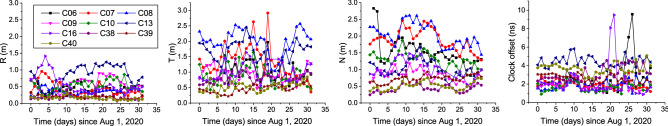
Orbit and clock offset accuracy variations of the IGSO satellites. From left to right: Radial, tangential, normal, satellite clock offset.

**Figure 4:**
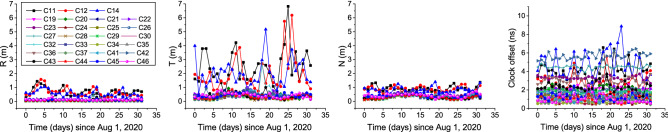
Orbit and clock offset accuracy variations of the MEO satellites. From left to right: Radial, tangential, normal, satellite clock offset.

**Figure 5:**
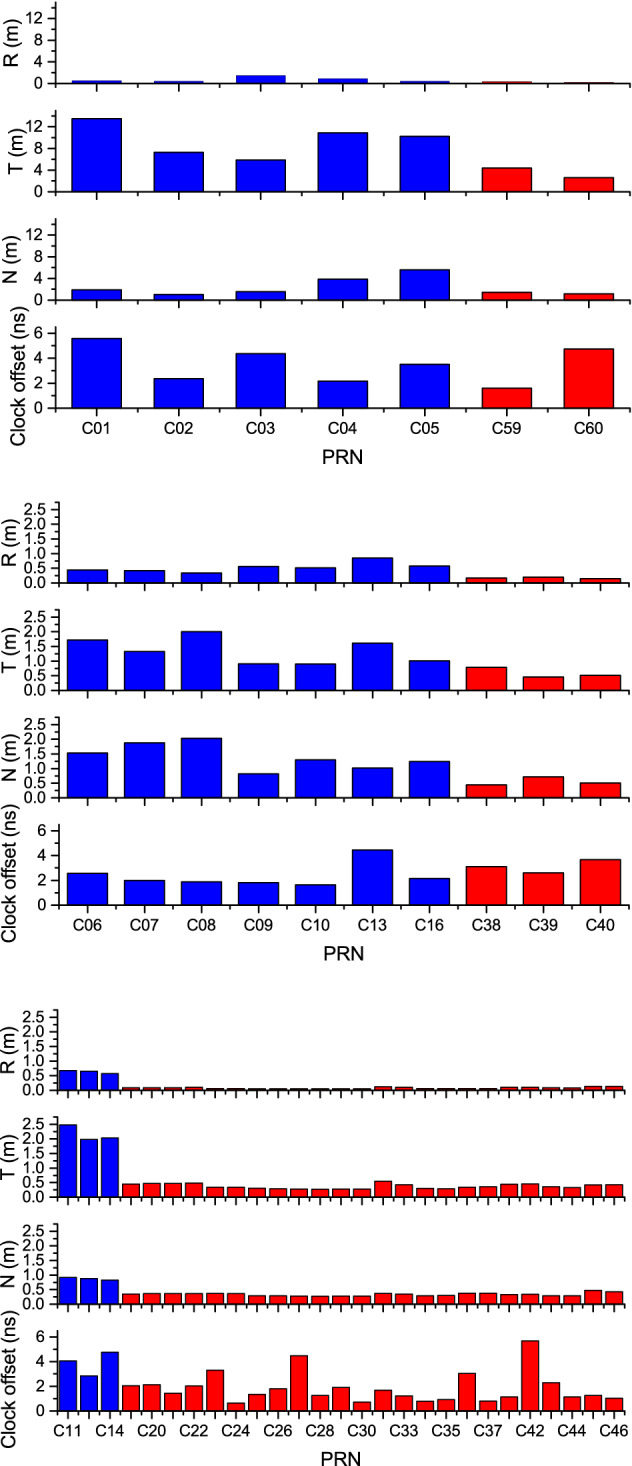
Comparison of BeiDou orbit and clock offset accuracies for three categories of GEO (top), IGSO (middle), and MEO (bottom). The blue and red bars denote BDS-2 and BDS-3 satellites, respectively.

The data from August 1 to September 1, 2020, a month after the official launching of the BDS-3 service, were considered. According to the three satellite categories of GEO, IGSO, and MEO, the Root Mean Square (RMS) of the errors in all the epochs in a day was considered as the statistical accuracy of the orbit and the clock offset. The orbit accuracy (radial, R; tangential, T; normal, N) and the satellite clock offset accuracy variations were taken into account for all the in-orbit operational satellites of BDS-2 and BDS-3, as illustrated in Figs. 2, 3, and 4. To further compare the accuracy, the average RMSs of the R, T, or N orbit error and the satellite clock offset error of each day in the counting period were considered for each satellite, as highlighted in Fig. 5. In Table 4, the statistical results of orbit and clock offset accuracies are averaged for all the BDS-2 and BDS-3 satellites of each category.

**Table 4:** Statistics of BeiDou orbit and clock offset accuracies. The results are derived from the averages of the corresponding values for all the same category of BDS-2 and BDS-3 satellites in Fig. 5, respectively.

	GEO		IGSO		MEO	
	BDS-2	BDS-3	BDS-2	BDS-3	BDS-2	BDS-3
R(m)	0.74	0.27	0.53	0.17	0.63	0.08
T(m)	9.57	3.51	1.26	0.59	2.16	0.37
N(m)	2.81	1.32	1.37	0.56	0.88	0.34
Clock offset(ns)	3.60	3.17	2.35	3.13	3.88	1.83

The following can be seen from the above results:

1. Compared to the BDS-2 GEO satellites, the BDS-3 GEO satellites of C59 and C60 exhibited significantly better orbit accuracies in R, T, and N directions. In terms of satellite clock offset accuracy, the C59 satellite was superior to the BDS-2 satellites, while the C60 satellite demonstrated a relatively lower clock offset accuracy. Upon examination, it was concluded that C60 was launched in March 2020 while C59 was launched in December 2018, due to which the C60 satellite clock was possibly still in the process of aging and due for further improvement in its performance^29^. On average, the R-, T-, and N-direction orbit accuracy and the satellite clock offset accuracy of the BDS-3 GEO satellites achieved 0.27 m, 3.51 m, 1.32 m, and 3.17 ns, respectively, an improvement of 63.5%, 63.3%, 53.0%, and 11.9% compared to that of BDS-2.
2. The R-, T-, and N-direction orbit accuracy of the BDS-3 IGSO satellites were remarkably higher than that of the BDS-2. However, the three IGSO satellites were all newly launched in 2019, not exhibiting evident advantages in terms of the satellite clock offset accuracy, similar to the case of the C60 satellite. On average, the R-, T-, and N-direction orbit accuracy of the BDS-3 IGSO satellites were 0.17 m, 0.59 m, and 0.56 m, respectively, which was improved by 67.9%, 53.1%, and 59.1%, respectively, compared to that of the BDS-2. The satellite clock offset accuracy reached 3.13 ns, which was marginally inferior to that of the BDS-2 IGSO satellites.
3. The BDS-3 MEO satellites displayed higher R-, T-, N-direction orbit accuracies than the BDS-2. They also delivered a comparatively steady and sound performance in terms of the satellite clock offset accuracy, except for a few of them (e.g., the C42 satellite launched in December 2019). On average, the R-, T-, and N-direction orbit accuracy and the satellite clock offset accuracy of BDS-3 MEO satellites were 0.08 m, 0.37 m, 0.34 m, and 1.83 ns, respectively, illustrating a significant improvement of 87.3%, 82.9%, 61.4%, and 52.8%, respectively, compared to that of BDS-2.

**Figure 6:**
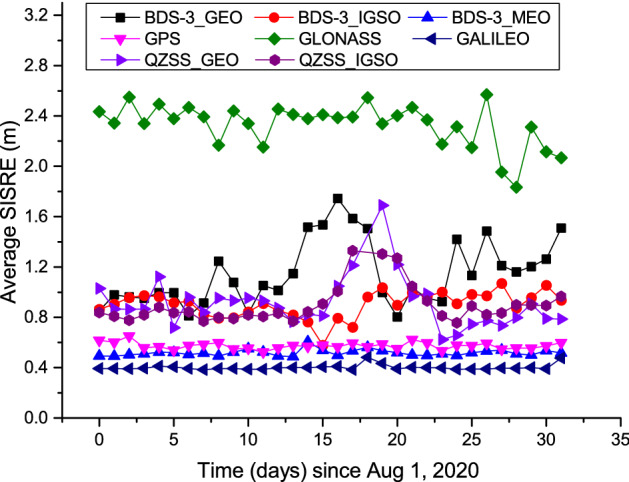
SISREs of major systems from Aug 1, 2020 to Sep 1, 2020. For comparison, BDS-3 satellites were grouped into the three categories of GEO, IGSO, and MEO. QZSS satellites are also made a similar classification.

To further compare the SISRE between BDS-3 and other systems, the analysis data above were also used. Initially, the SISRE of each epoch for every BDS-3 satellite was calculated. Then, the satellites were grouped into the three categories of GEO, IGSO, and MEO, and had the SISRE averaged in each group for all the epochs of each day. Besides, the same accuracy statistics were run for the SISRE of GPS, GLONASS, Galileo, and QZSS, to perform a better comparison. Moreover, QZSS included one GEO satellite and three IGSO satellites at the time, which were counted separately as in the case of BDS-3. As for the other systems, only MEO satellites were involved, thus demanding no categorization. Figure 6 illustrates the daily statistical result of SISRE during the analysis period for each system, while Table 5 enlists the average.

**Table 5:** Average SISRE for major systems. The results of different systems are the average of the SISREs for all days from Aug 1, 2020 to Sep 1, 2020.

System	SISRE(m)
BDS-3_GEO	1.15
BDS-3_IGSO	0.90
BDS-3_MEO	0.52
GPS	0.59
GLONASS	2.33
Galileo	0.40
QZSS_GEO	0.95
QZSS_IGSO	0.92

The following can be obtained from the results highlighted in Fig. 6 and Table 5:

1. The BDS-3 GEO, IGSO, and MEO satellites displayed an average SISRE of 1.15 m, 0.90 m, and 0.52 m, respectively.
2. Among the four primary global satellite navigation systems, when only the MEO satellites were considered, Galileo introduced the best space signal accuracy with an average SISRE of 0.40 m. Following Galileo, BDS-3 and GPS showcased an average SISRE of 0.52 m and 0.59 m, respectively. GLONASS demonstrated the worst performance with an average SISRE of merely 2.33 m.
3. The average SISRE of the BDS-3 IGSO satellites reached 0.90 m, which was on par with that (0.92 m) of the QZSS IGSO satellites. However, the average SISRE of the QZSS GEO satellites reached 0.95 m, which was better than the value of 1.15 m for the BDS-3 GEO satellites. Considering that the BDS-3 C60 satellite was still new since its launch and has room for improvement in its satellite clock offset accuracy, this situation can be treated as normal. In the future, the accuracy may be further enhanced with the progressive service provided by the C61 satellite.

### Analysis of positioning accuracy

**Figure 7:**
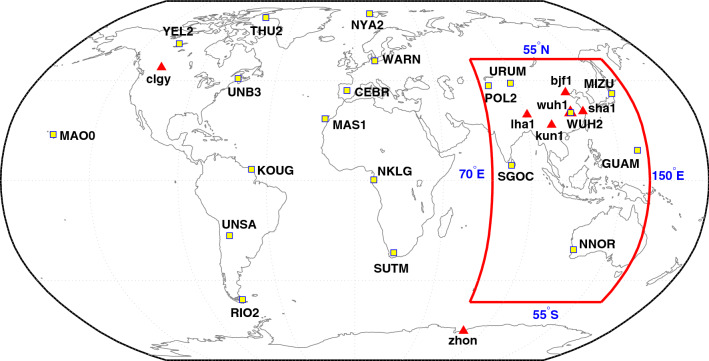
Distribution map of the observation stations. A red box is drawn to highlight the key BDS service area (55° S–55° N, 70° E–150° E). The MGEX station names are in the capital letters and the iGMAS station names are in the lowercase letters, which are also respectively labeled as ‘yellow square’ and ‘red triangle’.

To enhance the presentation of the analysis result of the positioning accuracy, when selecting the analysis data, the spatial and temporal coverage was fully considered. In terms of spatial coverage, 27 multi-system observation stations distributed globally were chosen, as demonstrated in Fig. 7. Among them, 20 MGEX observation stations and seven iGMAS (international GNSS monitoring and assessment service) observation stations were included, respectively labeled as ‘yellow square’ and ‘red triangle’. For convenient comparison, a red box is drawn in Fig. 7 that highlights the key BDS service area (55° S–55° N, 70° E–150° E). Moreover, to improve the temporal coverage of the analysis, from the days after the official service launch of the system, the observation data were chosen on the 1st date of August, September, October, November, and December in the year 2020, and on January 1, 2021, with a sampling interval of 30 s. Later, BDS-3 SPP and PPP processing were conducted for the data collected on these six days. The results were further compared with the known coordinates to evaluate the positioning accuracy.

### SPP accuracy

This section mainly analyzes SPP accuracy of BDS-3 in the two cases of single-frequency SPP and double-frequency SPP. In the former case, the single-frequency signals including B1C, B2a, B1I and B3I will be considered, and in the latter case, the double-frequency signals including B1C + B2a and B1I + B3I will be focused on.

1. Single-frequency SPP

The open positioning and navigation service signals of BDS-3 comprise the new signals of B1C and B2a along with the transitional signals of B1I and B3I. The former is only broadcast by the BDS-3 MEO and IGSO satellites, whereas the latter is broadcast by all the satellites of BDS-3 and BDS-2 category, as mentioned in Table 2. To assess the positioning performance of the signals, the observed pseudo-code for each single-frequency signal was utilized for SPP in each epoch. The ionospheric delay error is corrected by Klobuchar model. The satellite position and clock offset are calculated from the broadcast ephemeris. The least square estimation is used for user location estimation.

**Figure 8:**
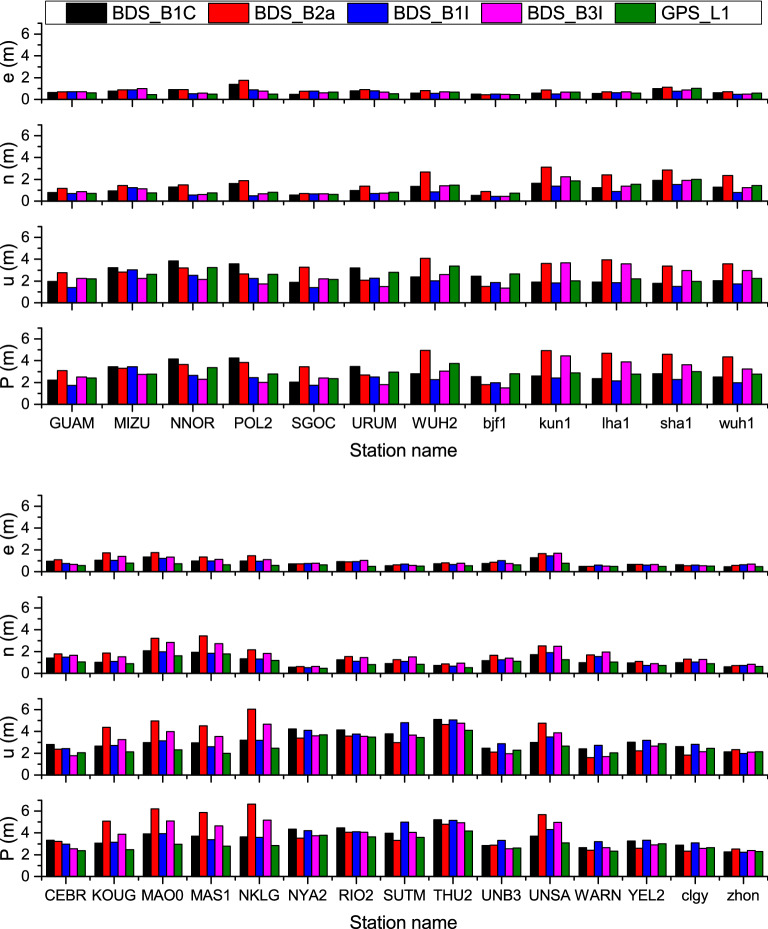
RMS statistics of single-frequency SPP positioning errors for stations in the Asian-Pacific region (top) and other regions (bottom). The MGEX station names are in the capital letters and the iGMAS station names are in the lowercase letters.

The RMSs of the east (e), north (n), up (u), and three-dimensional positioning errors were given in Fig. 8. For better comparison, the single-frequency positioning of the GPS L1 C/A code is also displayed in Fig. 8. Considering this, the positioning error RMS of each direction was averaged for all the observation stations, the results for which are enlisted in Table 6. It must be noted that single-frequency SPP has a wide range of application situations, the accuracy of which constitutes a key performance factor in the system construction.

**Table 6:** Average of the positioning errors RMS over all observation stations for single-frequency SPP (unit: m).

Signal	e		n		N		P	
	Asia–Pacific region	Other region	Asia–Pacific region	Other region	Asia–Pacific region	Other region	Asia–Pacific region	Other region
BDS_B1C	0.73	0.84	1.18	1.17	2.51	3.16	2.93	3.55
BDS_B2a	0.89	1.02	1.86	1.72	3.07	3.45	3.78	4.08
BDS_B1I	0.66	0.86	0.85	1.22	1.97	3.26	2.30	3.66
BDS_B3I	0.69	0.91	1.11	1.60	2.43	3.15	2.80	3.74
GPS_L1	0.60	0.59	1.12	0.99	2.51	2.67	2.88	2.97

The following can be concluded from Fig. 8 and Table 6:

1. Whether it was the Asian-Pacific region or other regions, the three-dimensional accuracy of single-frequency SPP for each signal frequency of BeiDou has always been better than 5 m on the whole. The east or north positioning result was particularly superior to that of the up direction. For all the four frequencies of BeiDou, the positioning accuracy was higher in the Asian-Pacific region than in other regions, since more satellites were visible in Asia–Pacific region because of the special BDS constellation.
2. Compared to the GPS L1 C/A code, the BDS-3 B1C, B2a, B1I, and B3I signals could achieve a comparable single-frequency SPP result. In the Asian-Pacific region, the three-dimensional positioning accuracies of B1I and B3I were comparatively better, achieving 2.30 m and 2.80 m, respectively, which were better than 2.88 m of GPS. In other regions, the three-dimensional positioning accuracy of B1C was found to be 3.55 m, which was the best among the four BeiDou signals, though marginally worse than 2.97 m of GPS. The causes for the phenomena above are as follows. B1I and B3I are transitional signals that are broadcast by both BDS-2 and BDS-3, which have an obvious advantage in the number of visible satellites in the Asian-Pacific region, thereby delivering higher positioning accuracy over there. In other regions, with only three BDS-2 MEO satellites, B1I and B3I do not possess a superior number of visible satellites. On the other hand, as a newly designed signal and being compatible with GPS L1 and Galileo E5, B1C possesses a significant advantage in the performance of capturing and tracking. However, it is the 24 BDS-3 MEO satellites that broadcast the B1C signal in other regions, which are still exceptionally fewer than the 31 in-orbit functioning satellites of GPS^30^. This is also the chief reason for its positioning accuracy being slightly lower than that of GPS.
3. Dual-frequency SPP

**Figure 9:**
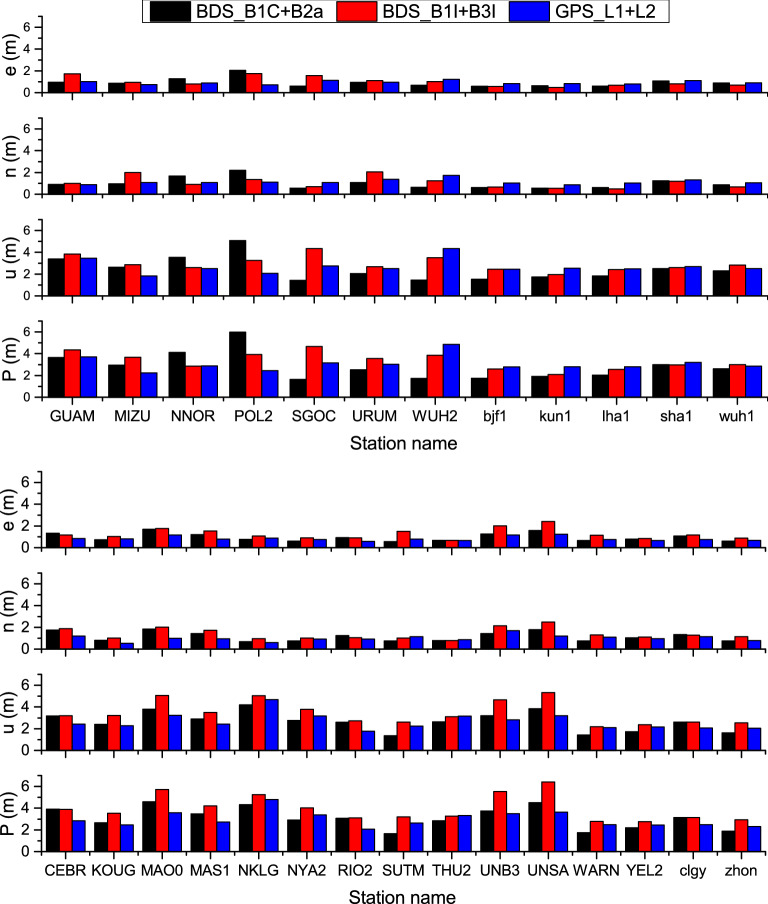
RMS statistics of dual-frequency SPP positioning errors for stations in the Asian-Pacific region (top) and other regions (bottom). The MGEX station names are in the capital letters and the iGMAS station names are in the lowercase letters.

Here, dual-frequency SPP processing was executed for the two dual-frequency pseudo-code combinations, namely B1C + B2a and B1I + B3I. The ionospheric delay error is corrected by using dual-frequency combination observation. The satellite position and clock offset are calculated from the broadcast ephemeris. The least square estimation is used for user location estimation. The output was compared with the L1 + L2 dual-frequency pseudo-code SPP result of GPS. The statistical approach for the accuracy was the same as that of the single-frequency SPP. The RMSs of the east (e), north (n), up (u), and three-dimensional positioning errors were given in Fig. 9. The positioning error RMS of each direction was averaged for all the observation stations, the results for which are enlisted in Table 7.

**Table 7:** Average of positioning errors RMS over all observation stations for dual-frequency SPP (unit: m).

Signal	e		n		u		P	
	Asia–Pacific region	Other region	Asia–Pacific region	Other region	Asia–Pacific region	Other region	Asia–Pacific region	Other region
BDS-3_B1C + B2a	0.80	0.80	0.82	0.99	2.09	2.27	2.39	2.63
BDS-3_B1I + B3I	0.69	0.73	0.76	1.15	2.48	2.90	2.70	3.34
GPS_L1 + L2	0.85	0.73	1.00	0.95	2.51	2.37	2.83	2.67

The following can be observed from Fig. 9 and Table 7.

1. BeiDou B1C + B2a and B1I + B3I, the two dual-frequency SPP schemes, exhibited relatively high accuracy in the Asian-Pacific region. Moreover, whether in Asia–Pacific or other regions, B1C + B2a dual-frequency positioning was significantly superior to B1I + B3I. Although more satellites broadcast B1I and B3I signals in Asia–Pacific, the more the distance between the two frequencies in dual-frequency positioning, the better the correction of ionospheric delay after their combination. In this regard, B1C + B2a is more advantageous since its positioning result was still better than that of B1I + B3I, even with fewer satellites.
2. In the Asian-Pacific region, the three-dimensional accuracy of BeiDou B1C + B2a dual-frequency SPP reached 2.39 m, marginally better than 2.83 m of GPS L1 + L2. In other regions, the three-dimensional accuracy of BeiDou B1C + B2a dual-frequency SPP reached 2.63 m, slightly better than 2.67 m of GPS L1 + L2. Considering the accuracy of the pseudo-code, it could be deemed that the two were fundamentally equal.

### PPP accuracy

**Figure 10:**
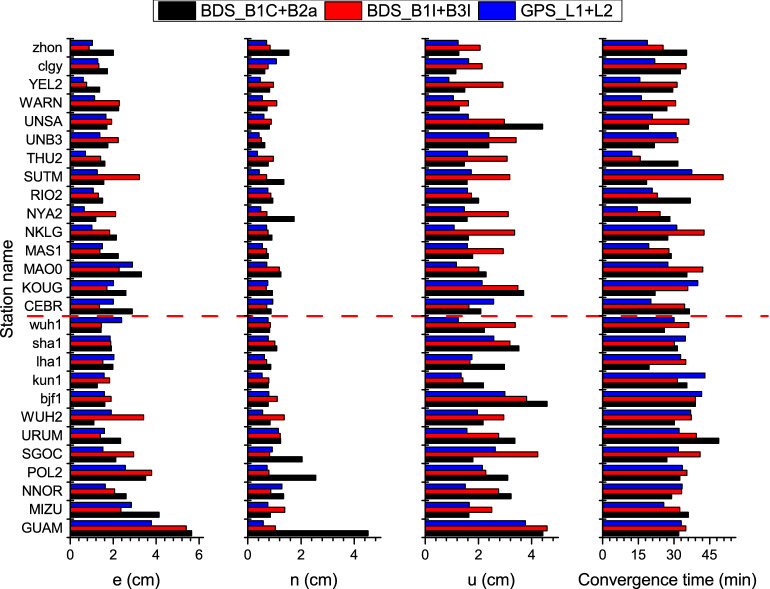
RMS statistics of dual-frequency PPP positioning errors and convergence time for stations in the Asian-Pacific region (below the red dash line) and other regions (above the red dash line). The MGEX station names are in the capital letters and the iGMAS station names are in the lowercase letters.

The two dual-frequency pseudo-code and carrier phase combinations of B1C + B2a and B1I + B3I were adopted here. Utilizing the same observation data from the stations in the Fig. 7, the PPP experiment for BeiDou was conducted, followed by computing the positioning accuracy after convergence. The ionospheric delay error is corrected by using dual-frequency combination observation. The satellite position and clock offset are calculated from the GBM precise orbit and clock offset product. The Kalman filtering is used for user location estimation. For convenient comparison, GPS L1 + L2 dual-frequency PPP was conducted at the same time. The convergence standard was set as when the e-, n-, and u-direction deviations were all lesser than 10 cm for more than 20 epochs^31^. The PPP convergence time was also computed for each processing mode. The above results are mentioned in Fig. 10. The results below the red dash line are from the Asian-Pacific observation stations, while the ones above are from the stations in other regions.

According to the comparison of the results in Fig. 9, both the new signal of B1C + B2a and the transitional signal of B1I + B3I could provide PPP results with centimeter accuracy to the observation stations globally. The Asian-Pacific region, though with a larger number of visible satellites, did not exhibit any obvious advantage. This is because the more visible satellites included some GEO satellites that had marginally lower precise orbit and clock offset accuracies, failing to improve the PPP accuracy significantly^32^. Hence, the observation stations here were not divided according to their location but considered altogether. The RMSs of e-, n-, and u-direction positioning errors were counted for all the observation stations in different positioning modes, as mentioned in Table 8. The following can be inferred from the above results.

1. After the convergence of B1C + B2a, the e-, n-, and u-direction accuracies achieved 2.2 cm, 1.2 cm, and 2.4 cm, respectively, on par with the processing results of B1I + B3I; though slightly worse than that of GPS L1 + L2. The accuracy of BeiDou PPP could be further improved due to the evolution of future precise products.
2. Except for a few observation stations, the PPP convergence time for BDS-3 B1C + B2a or B1I + B3I was fundamentally equal to that of the GPS L1 + L2, being approximately 30 min.

**Table 8:** Average of positioning errors RMS and convergence time over all observation stations for PPP.

Signal	e (cm)	n(cm)	u(cm)	Convergence time (min)
BDS3-3_B1C + B2a	2.2	1.2	2.4	30.3
BDS-3_B1I + B3I	2.1	0.9	2.7	33.8
GPS_L1 + L2	1.7	0.7	1.8	28.0

## Conclusions

In the present study, the SISRE computation method for different types of navigation satellites was present and the DCB correction method for BDS-3 new signals was deduced. In-depth analysis of the service accuracy levels have been analyzed from the perspective of BDS-3 SISRE and SPP or PPP positioning performance, leading to the following conclusions:

1. Compared to the BDS-2 satellites, the BDS-3 MEO, IGSO and GEO satellites exhibited significantly improved R, T, and N orbit accuracies. Except for a couple of newly launched satellites, the satellite clock offset accuracy was also remarkably enhanced.
2. The average SISREs of the BDS-3 MEO IGSO, and GEO satellites were 0.52 m 0.90 m and 1.15 m, respectively. Compared to the four major global satellite navigation systems consisting of MEO satellites, the SISRE of the BDS-3 MEO satellites was slightly inferior to 0.4 m of Galileo, slightly superior to 0.59 m of GPS, and remarkably superior to 2.33 m of GLONASS. The SISRE of BDS-3 IGSO was 0.90 m, which was on par with 0.92 m of QZSS IGSO. However, as the BDS-3 GEO satellites were newly launched and not completely functioning in orbit, their average SISRE was marginally worse than 0.91 m of the QZSS GEO satellites.
3. Single-frequency SPP of BDS-3 B1C, B2a, B1I, and B3I could all achieve remarkable positioning accuracy, with an overall three-dimensional positioning accuracy level better than 5 m. Among them, the B1I signal delivered the best positioning accuracy in the Asian-Pacific region while the B1C was leading in the other regions. Owing to the advantage in signal frequency, the dual-frequency SPP of B1C + B2a expressed better positioning accuracy compared to the transitional signal of B1I + B3I. The three-dimensional positioning accuracy levels for B1C + B2a of 2.39 m and 2.63 m were achieved respectively in the Asian-Pacific region and the other regions. Since there were more visible satellites in the Asia–Pacific, the positioning accuracy of BDS-3 was marginally better than that of GPS.
4. After convergence, the PPP accuracy of BDS-3 B1C + B2a or B1I + B3I was on the level of centimeters, slightly inferior to that of GPS L1 + L2. The convergence time, however, was similar for all three, which was approximately 30 min.

## Data Availability

All data generated or analyzed during this study are included in this published article.
